# Not all false positive diagnoses are equal: On the prognostic implications of false-positive diagnoses made in breast MRI versus in mammography / digital tomosynthesis screening

**DOI:** 10.1186/s13058-018-0937-7

**Published:** 2018-02-09

**Authors:** Christiane K. Kuhl, Annika Keulers, Kevin Strobel, Hannah Schneider, Nadine Gaisa, Simone Schrading

**Affiliations:** 1 Department of Diagnostic and Interventional Radiology, Hospital of the University of Aachen, RWTH, Pauwelsstrasse 30, 52074 Aachen, Germany; 2 Department of Pathology, Hospital of the University of Aachen, RWTH, Aachen, Germany

**Keywords:** Digital mammography, Digital breast tomosynthesis, Magnetic resonance imaging, Breast MRI, False-positive diagnoses, Atypia, Biopsy, Positive predictive value (PPV)

## Abstract

**Background:**

Breast magnetic resonance imaging (MRI) has been reported to frequently result in false-positive diagnoses, limiting its positive predictive value (PPV). However, for PPV calculation, all nonmalignant tissue changes are equally considered false-positive, although the respective prognostic importance, and thus patient management implications, of different pathologies may well differ. We investigated the pathology of false-positive diagnoses made by MRI compared with radiographic (digital mammography/tomosynthesis [DM/DBT]) screening.

**Methods:**

We conducted an institutional review board-approved prospective analysis of 710 consecutive asymptomatic women at average risk for breast cancer who underwent vacuum biopsy with or without surgical biopsy for screen-detected DM/DBT (*n* = 344) or MRI (*n* = 366) findings. We compared the frequency of false-positive biopsies (given by PPV3), as well as the types of nonmalignant tissue changes that caused the respective false-positive biopsies. In an order of increasing relative risk of subsequent breast cancer, pathologies of false-positive biopsies were categorized as nonproliferative, simple proliferative, complex proliferative, or atypical proliferative (including lobular carcinoma in situ/lobular intraepithelial neoplasia). The Mann-Whitney *U* test was used to compare distributions.

**Results:**

Histology yielded nonmalignant tissue in 202 of 366 biopsies done for positive MRI studies and 195 of 344 biopsies for positive DM/DBT studies, respectively, yielding a similar PPV3 percentages of 44.8% (164 of 202) and 43.3% (149 of 202) for both methods. However, the distribution of tissue types that caused false-positive diagnoses differed significantly (*p* < 0.0001). On the basis of MRI, high-risk atypical proliferative changes (40.1%; 81 of 202) were most common, followed by complex proliferative changes (23.8%; 48 of 202). In DM/DBT, low-risk, nonproliferative changes were the dominant reason for false-positive diagnoses (49.7%; 97 of 195), followed by simple proliferative changes (25.2%; 51 of 195). Low-risk nonproliferative changes resulted in false-positive diagnoses based on MRI as infrequently as did high-risk atypical proliferative changes based on DM/DBT (18.8% [38 of 202] vs. 18.0% [35 of 195]). The likelihood of a false-positive diagnosis including atypias was twice as high in women undergoing biopsy for MRI findings (81 of 202; 40%) as for those with DM/DBT findings (35 of 195; 18%).

**Conclusions:**

The prognostic importance, and thus the clinical implications, of false-positive diagnoses made on the basis of breast MRI vs. radiographic screening differed significantly, with a reversed prevalence of high- and low-risk lesions. This should be taken into account when discussing the rate of false-positive diagnoses (i.e., PPV levels of MRI vs. radiographic screening). Current benchmarks that rate the utility of breast cancer screening programs (i.e., cancer detection rates and PPVs) do not reflect these substantial biological differences and the different prognostic implications.

## Background

Breast magnetic resonance imaging (MRI) is used not only for diagnostic purposes and screening women at high risk of breast cancer but also is increasingly considered as a supplemental screening tool for women at average risk who have dense breast tissue [1–4]. In all these applications, MRI has yielded consistently higher sensitivity and increased cancer detection rates compared with digital mammography (DM).

Yet, breast MRI has frequently been reported to result in a large number of false-positive diagnoses. Such false-positive diagnoses add to the overall cost of screening because they necessitate additional workup by imaging or biopsy, may cause physical harm because of additional morbidity associated with biopsy procedures, and may cause emotional harm because they may generate breast cancer anxiety in the patient [5–9]. Accordingly, the reported high number of false-positive diagnoses has been a major reason for limiting the acceptance of breast MRI as a screening tool [10–12].

False-positive diagnoses in breast imaging are caused by a quite heterogeneous group of tissue changes. In principle, all positive diagnoses that prompt biopsy but yield nonmalignant tissues, including lobular carcinoma in situ/lobular intraepithelial neoplasia (LIN), are equally considered “false-positives.” Current guidelines distinguish between nonproliferative tissue changes (e.g., fibrocystic disease), proliferative tissue changes without atypias (e.g., usual ductal hyperplasia [UDH]), and proliferative changes with atypias (e.g., atypical ductal hyperplasia [ADH], atypical lobular hyperplasia [ALH], LIN) [13–16]. This classification of nonmalignant tissues has important clinical and prognostic implications. Whereas the presence of regressive, fibrocystic changes is associated with a slightly reduced risk of breast cancer, with a relative risk of 0.65 to 1.01, the relative risk increases by the 2-fold with the presence of proliferative tissue changes. It increases by 2.8-fold if there are multiple coexisting proliferative changes in the same biopsy [15–21] and even by 4- to 13-fold for proliferative changes that contain atypia [22–25]. Accordingly, for an average-risk woman undergoing screening for breast cancer, a (false-positive) diagnosis of atypical tissue changes does have important prognostic implications and will impact her further management [26–29]. This, however, is not reflected by the usual parameters that are used to benchmark the utility of screening programs (i.e., the cancer detection rate and positive predictive value [PPV]) [30].

The objective of this study was therefore to investigate the biological or prognostic significance of tissue changes that cause false-positive imaging diagnoses in MRI vs. radiographic breast imaging. Our aim was to provide a more differentiated, nuanced analysis of the clinical implications of so-called false-positive diagnoses. Because the prevalence of atypical or precancerous tissue changes depend on a woman’s risk of breast cancer, we took care to include only women without breast cancer-associated risk factors. To avoid bias secondary to underestimation or undersampling, the final analysis also included the results of all possible secondary excisional surgical biopsies and procedures done in the cohort.

## Methods

### Study setup and inclusion criteria

This prospective, institutional review board-approved cross-sectional study was conducted between June 2010 and January 2014 in an academic breast center. All patients provided written informed consent after the risks and benefits of the procedure had been thoroughly explained. We included all consecutive asymptomatic women at average risk who underwent mammography/digital breast tomosynthesis (DBT)-guided or MRI-guided biopsy during the study period.

*Asymptomatic* refers to the fact that we included only women whose biopsy was done for screening-detected imaging findings. *Average risk* refers to the fact we included only women without relevant breast cancer-associated risk factors such as a personal or family history of breast cancer, a prior tissue diagnosis with proliferative breast disease or atypias, or chest irradiation for Hodgkin’s disease.

This implies that women who had undergone DM/DBT alone, as well as women who had undergone supplemental MRI, for screening were included. Whether women underwent DM/DBT alone or DM/DBT plus MRI for screening depended on their breast tissue density and their willingness to undergo supplemental breast MRI screening.

MRI-guided biopsy is performed only in patients who have MRI-only visible findings. All women who had MRI had also had DM/DBT. Accordingly, all women who underwent MRI-guided biopsy had a negative DM/DBT at the site of the MRI finding. However, women who underwent DM/DBT-guided biopsy could have had (a) no MRI or (b) a negative MRI or (c) a positive MRI at the site of the DM/DBT abnormality requiring biopsy.

### Imaging and biopsy methods

All screening studies (DM/DBT and/or MRI) had been read and classified according to the Breast Imaging Reporting and Data System (BI-RADS) lexicon [31] by one of six breast radiologists with between 2 and 18 years of experience in reading mammograms and breast MRI scans. Independent double reading of screening mammograms/DBT scans was performed as per usual practice.

In the first half of the study period (2010–2011), women underwent mammographic screening by bilateral two-view full-field DM (Selenia Dimensions; Hologic, Marlborough, MA, USA). In the second half of the study period (2012–2013), women underwent two-view DBT in addition (Selenia Dimensions).

All MRI studies were performed on a 1.5-T system (Achieva; Philips, Amsterdam, The Netherlands) with a four-element breast coil (Open Breast Array Coil; Invivo, Gainesville, FL, USA) as described previously [3]. For screening, we used bilateral axial 2D multislice gradient echo imaging before and four times after bolus injection of 0.1 mmol/kg body weight gadobutrol (Bayer Healthcare, Whippany, NJ, USA) and a T2-weighted turbo spin echo pulse sequence with matching geometry.

Before DM/DBT- or MRI-guided biopsy was considered, women had undergone an appropriate diagnostic assessment including additional mammographic views and cone views. Only if, after this workup, the final diagnosis was BI-RADS 4 or 5, did the patient proceed to undergo mammographic (DM- or DBT–guided) vacuum biopsy or DM/DBT-guided needle localization [32]. Women underwent MRI-guided biopsy only if they had MRI findings that did not exhibit a correlate on DM/DBT studies.

DM/DBT or MRI-guided vacuum biopsy was performed in close adherence to national and international practice guidelines [33]. Vacuum biopsies or needle localizations for findings based on DM were performed on a dedicated prone stereotactic table (Lorad/MultiCare Platinum; Hologic) or under DBT guidance using the Affirm™ system (Hologic) as described previously [34]. All vacuum biopsies were performed with a 9-gauge device (Eviva). MRI-guided breast biopsies or needle localizations were performed using the DynaCAD system (Invivo) for biopsy planning and a 9-gauge biopsy device (ATEC).

### Histologic processing and classification of benign lesions

All biopsy specimens were processed according to a standard protocol based on recent European guidelines and were reviewed by a certified breast pathologist with more than 20 years of experience. If necessary for classification, further pathological assessment was performed at the discretion of the pathologist and included additional layering or immunohistochemical examination (e.g., cytokeratin 5/6 (CK5/6), CK14, CK17, E-cadherin, p63, Ki-67, smooth muscle actin).

A careful interdisciplinary radiologic-pathologic correlation was performed for each target lesion. If discordance between the imaging finding and the pathologic result occurred, the patient was recalled for repeat diagnostic imaging to check whether the target had been biopsied successfully. If doubt persisted, rebiopsy, usually as surgical excisional biopsy, was done. All women with a diagnosis of cancer (i.e., ductal carcinoma in situ [DCIS] or invasive cancer) and all women with a biopsy-based diagnosis of cellular atypias (flat epithelial atypia, ADH, ALH, LIN) underwent subsequent surgical excision. This was also performed for core biopsies that, according to the European Breast Cancer Screening Pathology Classification System, were categorized as B3 (uncertain biologic potential) [35].

In women undergoing surgery, the final surgical pathology report was used in addition to the core biopsy diagnosis for further analysis. For women who did not proceed to surgical resection, the needle biopsy tissue diagnosis was used. These latter women underwent follow-up to confirm lesion stability and thus rule out breast cancer for at least 18 months (range, 18–42 months).

All cases with nonmalignant breast pathology diagnoses—obtained by needle biopsy and/or by surgical excisional biopsy—were categorized according to the method of Dupont and Page [16] into one of the following groups: nonproliferative, proliferative without atypias, and proliferative with atypias. According to the method of Worsham et al. [17], the category proliferative without atypias was further subdivided into simple proliferative, where only one type of tissue proliferation was present, and complex proliferative, where more than one type of proliferative change was present.

Typical histological findings that were categorized as nonproliferative were apocrine and fibrocystic changes, inflammatory changes, fat necrosis, and fibroadenomas. Findings categorized as simple or complex proliferative were all types of adenosis, including sclerosing adenosis, UDH, papilloma without atypia, and complex sclerosing lesions. Findings categorized as proliferative with atypias were ADH, flat epithelial atypia, papilloma with atypia, and LIN.

### Data collection and analysis

The following data were prospectively recorded: family and personal history, history of prior breast biopsy, demographic data, imaging features of target lesions at DM/DBT and MRI, and final histological result. PPV 3 (PPV of biopsy) of MRI and DM/DBT were calculated considering only malignant lesions (invasive cancer and DCIS) as test-positive findings.

To avoid data clustering and risk confounders, all analyses were done on a per-patient basis; that is, we included only the dominant lesion in the analysis. The dominant lesion was identified on the basis of its therapeutic or prognostic implication as follows: nonproliferative < simple proliferative < complex proliferative < proliferation with atypias < DCIS or invasive cancer. This implies that a tissue diagnosis of proliferation with atypias identified in a patient who also had a diagnosis of DCIS or invasive cancer elsewhere in the same or the opposite breast was not included in the analysis of distribution of nonmalignant lesions. This also implies that in a patient with proliferation with atypias of any type, coexisting simple proliferative or complex proliferative tissue changes were not considered for further analysis.

We compared the distribution of the different categories (nonproliferative, simple proliferative, complex proliferative, atypical proliferative) using the Mann-Whitney *U* test. For all distributions, 95% Clopper-Pearson CIs were calculated. A two-sided *p* value ≤ 0.05 was considered statistically significant. All analyses were performed using IBM SPSS Statistics version 22.0 software (IBM, Armonk, NY, USA).

## Results

### Patients

During the study period, a total 710 consecutive asymptomatic women at average risk received the final DM/DBT or MRI diagnosis of a suspicious finding (BI-RADS 4 or 5) and thus underwent image-guided biopsy. Of these, 344 women aged 56.4 ± 10.8 years underwent DM/DBT guided biopsy, and 366 women aged 54.1 ± 10.4 years underwent MRI-guided biopsy. Of the 344 women who underwent biopsy for DM/DBT findings, 39 (11.9%) had also undergone MRI. In 5 (1.5%) of these 344 patients, the MRI finding was positive at the site of the DM/DBT abnormality. Because the finding was visualized by DM/DBT, patients proceeded with DM/DBT-guided biopsy. Of the 366 women who underwent biopsy for MRI findings, all had undergone DM/DBT, but none had a DM/DBT correlate for the MRI finding, and therefore they proceeded to MRI-guided biopsy.

Details on patient demographics and the distribution of breast densities are given in Table 1. Women who underwent MRI for screening tended to have somewhat denser breast tissue, with a distribution of nondense (American College of Radiology [ACR] A and B) vs. dense (ACR C and D) of 56.4% (194 of 344) vs. 43.6% (150 of 344) for DM/DBT, compared with 33.9% (124 of 366) vs. 66.1% (242 of 366) for MRI. Otherwise, the two groups were similar.

**Table 1:** Demographics and breast density distribution of the two cohorts

	DM/DBT (*n* = 344)	MRI (*n* = 366)
Patient age, years		
Mean ± SD	56.39 ± 10.77	54.12 ± 10.39
Median (range)	54 (37–80)	54 (22–87)
Menopausal status		
Premenopausal	20.1% (69 of 344)	23.8% (87 of 366)
Postmenopausal	79.9% (275 of 344)	76.2% (279 of 366)
Breast density		
ACR A	14.8% (51 of 344)	4.1% (15 of 366)
ACR B	41.6% (143 of 344)	29.8% (109 of 366)
ACR C	32.6% (112 of 344)	46.7% (171 of 366)
ACR D	11% (38 of 344)	19.4% (71 of 366)

*Abbreviations: ACR* American College of Radiology, *DBT* Digital breast tomosynthesis, *DM* Digital mammography

### Histological results

A similar rate of benign lesions (i.e., rate of false-positive diagnoses) was observed for DM/DBT (195 of 344 [56.7%]; 95% CI, 51.3–62.0%) and MRI (202 of 366 [55.2%]; 95% CI, 49.9–60.4%); accordingly, PPV3 was similar (Table 2).

**Table 2:** Positive predictive value (PPV3) for digital breast tomosynthesis/digital mammography and magnetic resonance imaging

	DM/DBT	MRI
Percent	43.3%	44.8%
No. of patients	149 of 344	164 of 366
95% CI	38.0–48.7%	39.6–50.1%

*Abbreviations: DBT* Digital breast tomosynthesis, *DM* Digital mammography, *MRI* Magnetic resonance imaging

The histopathological tissue changes that had caused the false-positive diagnoses, however, differed significantly between DM/DBT and MRI (*p* < 0.0001). In summary, the distributions of tissue categories was more or less reversed (Table 3).

**Table 3:** Distribution of tissue categories found in nonmalignant breast biopsies

	DM/DBT (*n* = 195)			DCE-MRI (*n* = 202)		
Type	No. of patients	%	95% CI	No. of patients	%	95% CI
Nonproliferative	97	49.7%	42.5–57.0	38	18.8%	13.7–34.9
Simple proliferative	51	25.2%	20.1–32.9	35	17.3%	12.4–23.3
Complex proliferative	12	6.2%	3.2–10.5	48	23.8%	18.1–30.1
Proliferative with atypias	35	18.0%	12.8–24.1	81	40.1%	33.3–47.2
Nonproliferative or simple proliferative	148	75.9%	69.3–81.7	73	36.1%	29.5–43.2
Complex or atypical proliferative	47	24.1%	18.3–30.7	129	63.9%	56.8–70.5
All	195	100.0%		202	100.0%	

*Abbreviations: DBT* Digital breast tomosynthesis, *DCE* Dynamic contrast-enhanced imaging, *DM* Digital mammography, *MRI* Magnetic resonance imaging

**Fig. 1:**
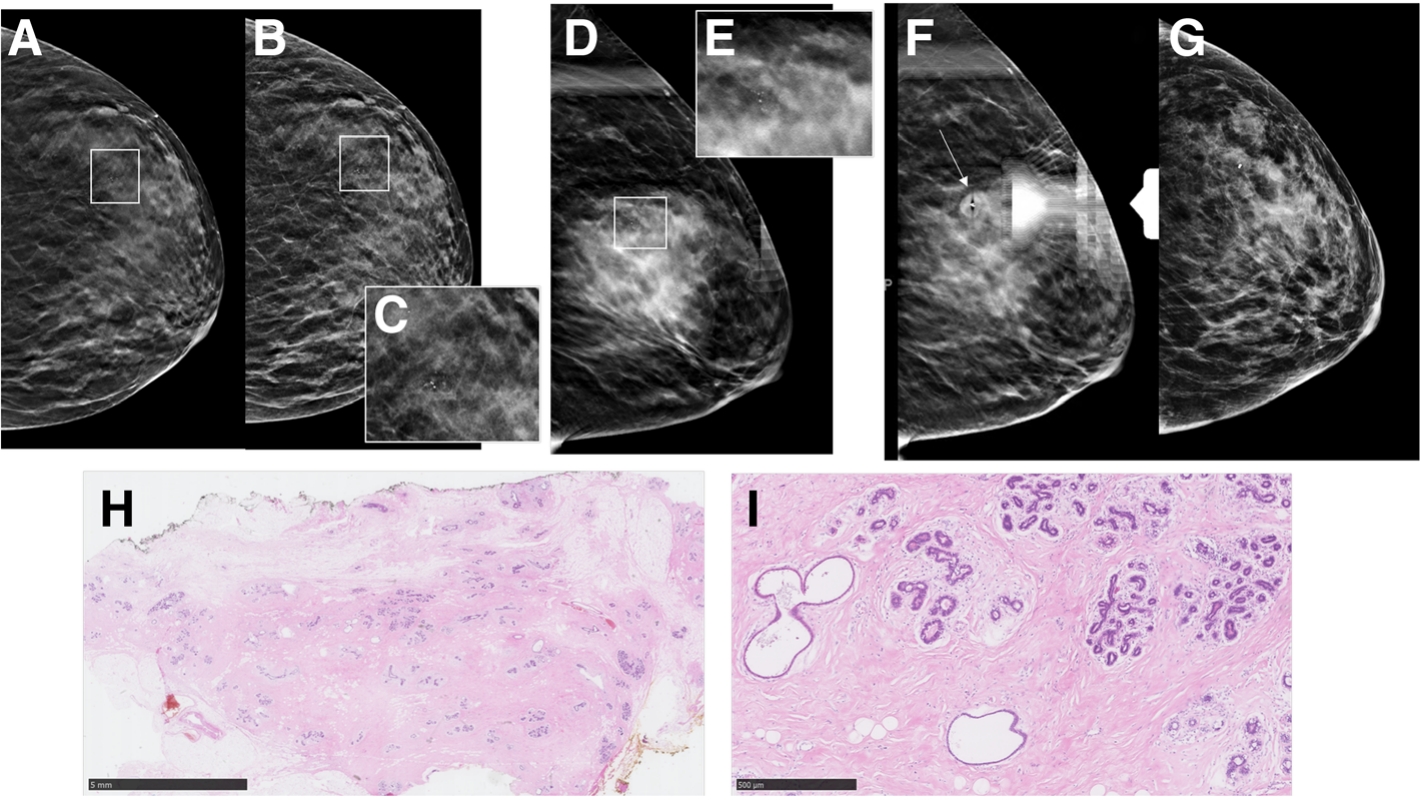
False-positive digital mammography (DM)/digital breast tomosynthesis (DBT) screening diagnosis. Screening DBT (**a**) with reconstructed 2D DM (C-view) (**b**) and higher-magnification view (**c**) in a 55-year-old woman at average risk revealed clustered calcifications in the left upper outer quadrant. DBT was rated as Breast Imaging Reporting and Data System 4, and DBT-guided vacuum biopsy was performed (**d**–**g**). Histology revealed fibrocystic changes with sclerosing adenosis and no atypia (**h** and **i**). Overview biopsy specimen (**h**) and higher-magnification view (**i**) of H&E stains. No immunohistochemistry was necessary. **a**–**c** Screening DBT (**a**) with reconstructed 2D DM (C-view) (**b**) and higher-magnification view (**c**). **d**–**g** DBT-guided vacuum biopsy was performed, including clip placement. **h** and **i** Overview biopsy specimen (**h**) and higher-magnification view (**i**) of H&E stains. No immunohistochemistry was necessary

**Fig. 2:**
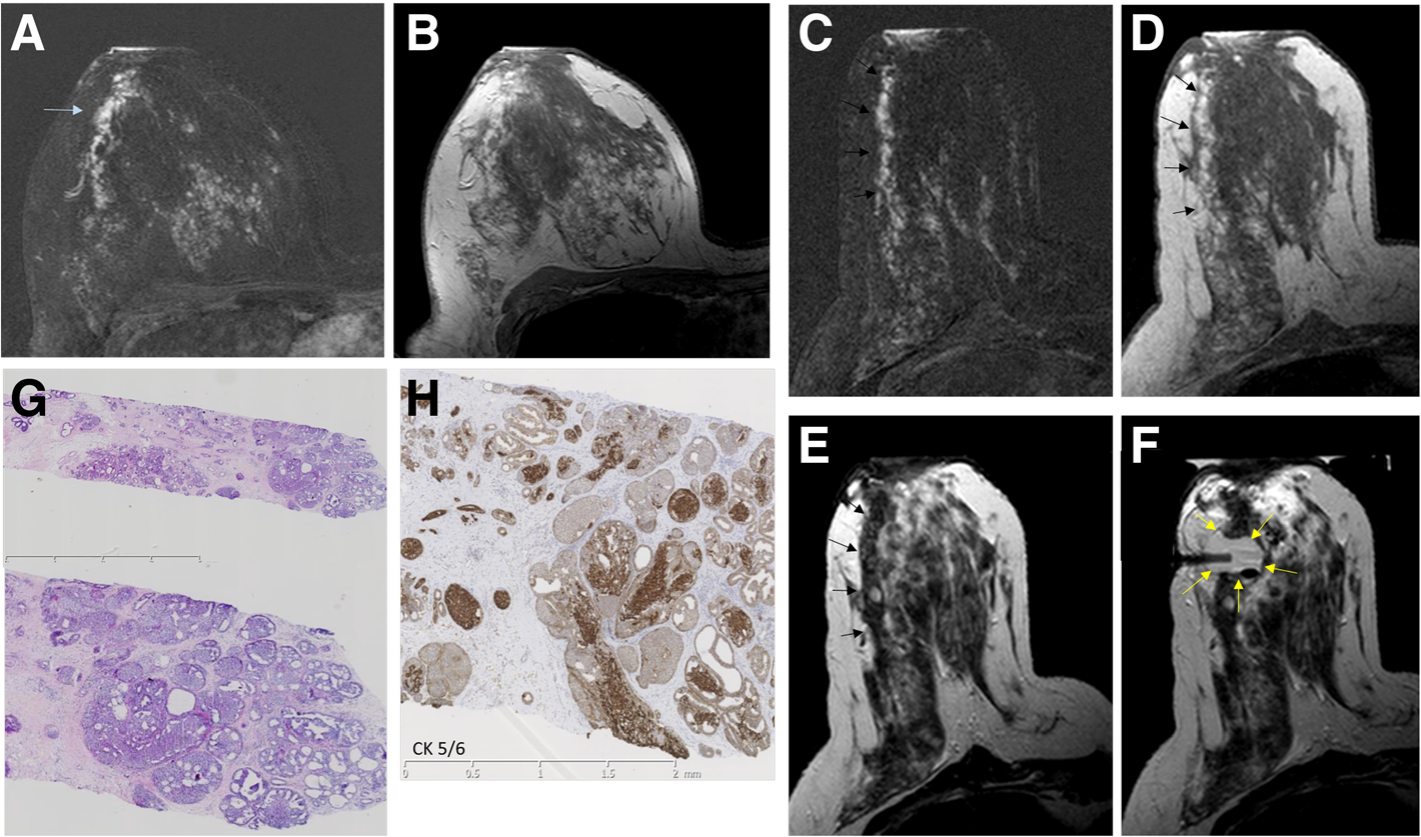
False-positive magnetic resonance imaging (MRI) screening diagnosis. Screening breast MRI (**a** and **b**) in a 51-year-old woman at average risk undergoing screening MRI. MRI showed moderate background enhancement (American College of Radiology C) and a nonmass enhancement with segmental distribution in the right breast (*arrow* in **a**). MRI-guided vacuum biopsy was performed (**c**–**f**). Histology revealed atypical ductal hyperplasia (ADH), flat epithelial atypia, and sclerosing adenosis (**g**). Immunohistochemical staining was needed to confirm ADH (**h**). The patient subsequently underwent open surgery, which confirmed the presence of ADH; no ductal carcinoma in situ or invasive cancer was found. The patient has been in follow-up, including serial MRI, for 2 years, so far without a diagnosis of invasive cancer. **a** and **b** Screening diagnostic breast MRI. First postcontrast subtracted (**a**) and nonsubtracted images (**b**). **c**–**f** MRI-guided vacuum biopsy of the segmental nonmass enhancement in the right breast (*black arrows* in **d**). First postcontrast subtracted (**c**) and nonsubtracted images (**d**), T2-weighted turbo spin echo (TSE) before (**e**) and after (**f**) biopsy with the biopsy cavity, which demonstrates successful biopsy (*yellow arrow*). **g** and **h** Histology after H&E staining (**g**) and immunohistochemical staining with cytokeratin 5/6 (CK5/6) (**h**). **a** and **b** Screening diagnostic breast MRI. First postcontrast subtracted (**a**) and nonsubtracted image (**b**). **c**–**f** MRI-guided vacuum biopsy of the segmental nonmass enhancement in the right breast (*black arrows* in **d**). First postcontrast subtracted (**c**) and nonsubtracted images (**d**), T2-weighted TSE before (**e**) and after (**f**) biopsy with the biopsy cavity, which demonstrates successful biopsy (*yellow arrow*). **g** and **h** Histology after H&E staining (**g**) and immunohistochemical staining with CK5/6 (**h**)

**Fig. 3:**
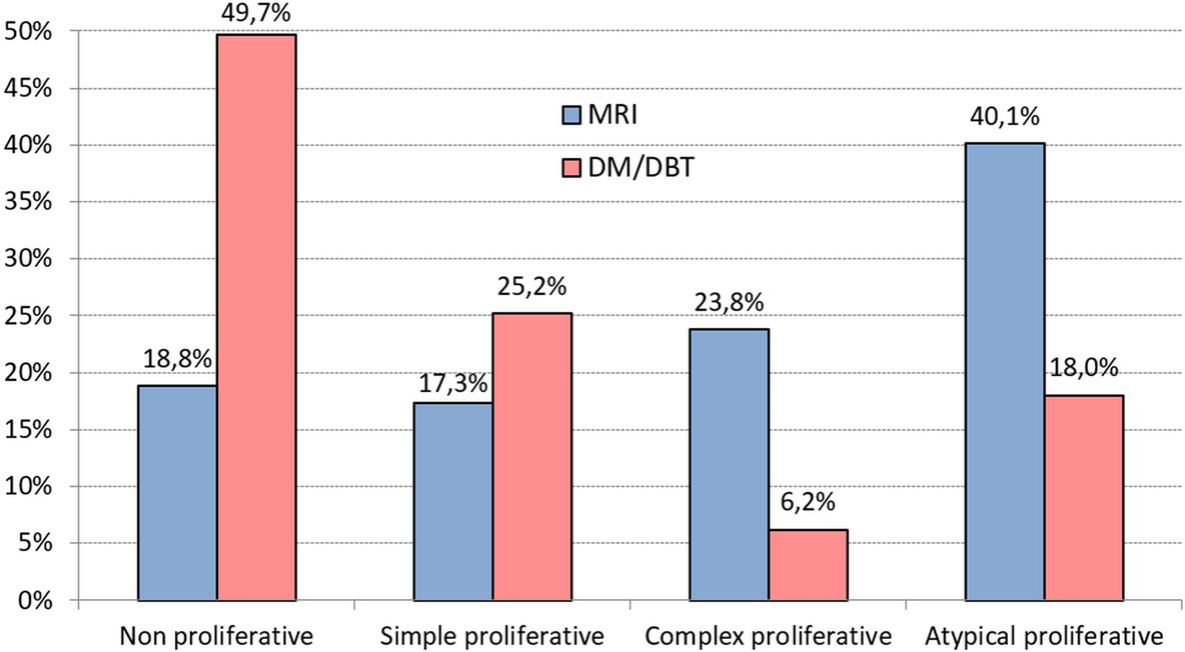
Distribution of tissue changes causing false-positive finding based on magnetic resonance imaging (MRI) vs. digital mammography/digital breast tomosynthesis (DM/DBT)

The major reason for false-positive diagnosis based on DM/DBT (97 of 195; 49.7%) were nonproliferative changes (Figs. 1 and 2). By way of contrast, the major reason for false-positive diagnosis based on MRI were proliferative changes with atypias (81 of 202; 40.1%). Nonproliferative changes were a rare cause of false-positive diagnosis based on MRI (38 of 202; 18.8%); similarly rare was proliferation with atypias as a cause of false-positive diagnosis based on DM/DBT (35 of 195; 18.0%). Thus, the likelihood of a false-positive diagnosis containing atypias was twice as high in women undergoing biopsy for MRI-based findings (81 of 202; 40%) as for DM/DBT-based findings (35 of 195; 18%) Fig. 3.

The second most important reason for false-positive diagnosis based on MRI was complex proliferative tissue changes (48 of 202; 23.8%), whereas with DM/DBT, the second most important reason was simple proliferative changes (51 of 195; 25.2%). The five patients with positive MRI findings at the site of the mammographic abnormality all had proliferative tissue changes with (*n* = 3) and without (*n* = 2) atypias.

## Discussion

In this study of 710 women undergoing biopsy for screen-detected DM/DBT and MRI findings, we found that the rate of true- over false-positive diagnoses (i.e., the PPV) was similar, with 43.3% for DM/DBT and 44.8% for MRI. However, the biological or prognostic significance of tissue changes that caused the respective false-positive diagnoses was clearly dissimilar. Whereas nonproliferative, regressive changes were the single most important cause of false-positive diagnoses based on radiographic breast imaging (97 of 195; 49.7%) (i.e., tissues that do not modulate a woman’s risk of subsequent breast cancer), the single most important cause of false-positive MRI diagnoses (81 of 202; 40.1%) was atypical proliferation. Nonproliferative tissue changes were as rarely seen to cause false-positive diagnoses based on MRI as were atypical proliferative tissue changes seen to cause false-positive diagnoses based on DM/DBT (18.8% [38 of 202] vs. 18.0% [35 of 195]).

The benchmarks used for auditing breast cancer screening efficacy are the respective programs’ cancer detection rates and the PPVs [30]. The PPV is a measure that describes the power with which an imaging method is able to classify findings. To calculate the PPV for breast cancer screening programs, all positive imaging findings that are caused by invasive cancer or DCIS are considered “true-positives,” whereas all positive imaging findings that are proven to be caused by anything but cancer or DCIS are considered “false-positives.”

For breast MRI screening, although the cancer detection rate is consistently higher than that achievable with mammographic or ultrasound screening, the high number of false-positive diagnoses (i.e., the low PPVs) that have been reported limit the perceived net benefit of this screening method [36–39]. However, in keeping with more recent studies on contemporary breast MRI protocols [1, 40–42], our study demonstrates that screening with MRI can be done with PPVs that are indeed comparable to those achieved with DBT. Moreover, our results suggest that simply comparing numeric PPVs might yield a misleading risk-benefit assessment of different screening methods. A significant fraction of false-positive diagnoses made on the basis of breast MRI were due to high-risk lesions. Diagnosis of such tissue atypias in women who, on the basis of their personal and family histories, were considered to carry an average risk yields important information on the respective women’s future management.

It is well established that MRI-guided biopsies yield many high-risk tissue changes [43–47]. However, so far, breast MRI, and thus MRI-guided biopsy, has been reserved for women who carry a high lifetime risk of breast cancer or who undergo MRI because of clinical or imaging findings suspicious of breast cancer. Yet, the distribution of histological tissue types, especially the prevalence of atypical proliferation or of high-risk lesions in general, will greatly depend on the individual woman’s risk of breast cancer [13, 15, 16, 19]. Meaningful comparisons of the natural distribution of tissue changes in false-positive MRI- and DM/DBT-guided biopsies are possible only if the respective screening cohorts exhibit similar risk profiles. This is what our study offers; both cohorts (i.e., women undergoing biopsy for positive DM/DBT screening findings or for positive MRI screening findings) were asymptomatic and had no breast cancer-associated risk factors.

On pathological or pathophysiological grounds, it is plausible that the majority of false-positive diagnoses made on the basis of DM and DBT are due to regressive tissue changes. Microcalcifications, especially those caused by benign histological changes, usually represent regressive changes and are not correlated with cell proliferation [48]. In contrast, tissue changes leading to false-positive diagnoses based on MRI are depicted because of their contrast enhancement. Dynamic contrast enhancement in MRI does indeed correlate with tissue proliferation [49].

The association of atypical tissue changes with subsequent breast cancer has been consolidating over the past years. As evidenced by common patterns of genomic additions and deletions, breast cancer progression is a biological continuum, starting from normal breast tissue via flat epithelial atypia, ADH, to DCIS, and then to invasive breast cancer [50, 51]. Tissue changes with atypias such as ADH and ALH would thus not only mark an increased risk of breast cancer but also might represent true precursors of the actual breast cancer that may follow [52, 53]. In keeping with these concepts, recent evidence on the natural history of women diagnosed with ADH or ALH suggests that both histological types are associated with similar degrees of risk, indicating that it is the presence or absence of atypical cells, regardless of their morphology or presumed cell of origin, that drives the risk of subsequent breast cancer [22–25, 53]. All in all, it appears that the actual risk of subsequent invasive breast cancer is not substantially different for women diagnosed with ADH or ALH vs. low-grade DCIS [23, 53, 54].

Accordingly, we propose a careful discussion on what constitutes a true-positive vs. a “false-positive” diagnosis in women at average risk who undergo screening for breast cancer. If it is agreeable that a true-positive diagnosis should be one that has an impact on a woman’s further management (i.e., an “actionable” diagnosis), then diagnosis of atypical tissue changes could indeed be considered a true-positive diagnosis. Most practice guidelines recommend intensified surveillance, preventive surgery, or even chemoprevention for women with a tissue diagnosis of ADH, ALH, or LIN [26–29]. Regardless of how we label false-positive imaging diagnoses, breast radiologists should be aware of the fact that not all false-positive diagnoses are equal in that not all are only unwanted side effects of the desire to establish a diagnosis of breast cancer early, but many, especially many false-positive MRI diagnoses, provide valuable information that is helpful for guiding further patient management. Last, but especially important, we suggest that this information be given to women with dense breasts when they are counseled about the advantages and disadvantages of different supplemental breast cancer screening methods.

A possible limitation of our study is the fact that the breast density distribution of the study population that underwent MRI was slightly shifted toward those with denser breast tissue, in accordance with the clinical use of MRI for supplemental screening of women with dense breast tissue. However, it has previously been shown that breast density and rate of proliferative breast disease are not correlated [19]. The switch from pure DM to DM/DBT screening may have influenced our false-positive biopsy rate for radiographic screening. However, the PPV observed for DM/DBT in our cohort is within the range of PPVs expected for routine screening mammography, so the impact of switching of technology, if it exists, should not be very large.

One may argue that the MRI cohort was “enriched” for high-risk lesions because the lower-risk lesions were verified by DM/DBT. However, the null hypothesis of our study was that the distribution of low- and high-risk tissue changes is identical in women with DM/DBT-positive vs. MRI-positive screening findings. If the null hypothesis was to be accepted, then no enrichment would be possible, because high- and low-risk lesions would have been biopsied with similar frequency. Yet, our null hypothesis must be rejected; the distribution is indeed quite dissimilar. Accordingly, just because DM/DBT will indeed preferably pick up low-risk findings is an enrichment at all conceivable. Such enrichment would thus be a consequence of the very findings made in this study. Furthermore, the degree of enrichment is determined by the degree of overlap (i.e., the number of women who had suspicious findings on both MRI and DM/DBT). However, this number was small, with 1.5% of DM/DBT-positive findings also yielding MRI-positive results, all representing intermediate- or high-risk lesions based on histology. Accordingly, the effect of enrichment in our cohort is likely negligible.

## Conclusions

Our study does not merely confirm previous results on the similar PPVs (PPV3) of radiographic (DM/DBT) and breast MRI. We demonstrate that the prognostic relevance of the false-positive diagnoses that drive the respective PPVs differs depending on the imaging method, with high-risk lesions being the predominant cause of false-positive MRI diagnoses, as compared with low-risk lesions being the predominant cause of false-positive radiographic diagnoses. This should be considered when discussing the rate of false-positive findings in MRI vs. radiographic breast cancer screening and should be explained when counseling women about their choices and the risks and benefits of supplemental screening.

## Abbreviations

ACR: American College of Radiology
ADH: Atypical ductal hyperplasia
ALH: Atypical lobular hyperplasia
BI-RADS: Breast Imaging Reporting and Data System
CK: Cytokeratin
DBT: Digital breast tomosynthesis
DCE: Dynamic contrast-enhanced imaging
DCIS: Ductal carcinoma in situ
DM: Digital mammography
LIN: Lobular intraepithelial neoplasia
MRI: Magnetic resonance imaging
PPV: Positive predictive value
TSE: Turbo spin echo
UDH: Usual ductal hyperplasia
